# Pre- and peri-operative factors influence autogenous tooth transplantation healing in insufficient bone sites

**DOI:** 10.1186/s12903-021-01686-x

**Published:** 2021-06-29

**Authors:** Thanapon Suwanapong, Aurasa Waikakul, Kiatanant Boonsiriseth, Nisarat Ruangsawasdi

**Affiliations:** 1 Department of Oral and Maxillofacial Surgery, Faculty of Dentistry, Mahidol University, 6 Yothee st., Ratchathewi, Bangkok, 10400 Thailand; 2 Department of Pharmacology, Faculty of Dentistry, Mahidol University, 6 Yothee st., Ratchathewi, Bangkok, 10400 Thailand

**Keywords:** Autogenous tooth transplantation, Severely insufficient alveolar bone, Autotransplantation, Operative factors, Bone healing

## Abstract

**Background:**

The amount of bone remaining at the transplant site for autogenous tooth transplantation can facilitate successful healing. Therefore, this retrospective study evaluated the factors influencing the healing of 50 successful autogenous tooth transplantations with insufficient bone support at the transplanted site without a bone graft.

**Methods:**

The factors were classified as pre- and peri-operative factors, and the healing outcomes were clinical and radiographic observations. The factors were statistically analyzed using the chi-square test to identify correlations between the pre- and peri-operative factors and the clinical and radiographic outcomes. The T-test or one-way analysis of variance was used to compare the percent bone change in each factor.

**Results:**

The results indicated that gingival healing was delayed in the intra-arch transplantations, and pulp obliteration was seen earlier when transplanted in the maxilla. Patients under 18-years-old demonstrated a greater percent bone change than the over 18-year-old patients, while the peri-operative variables did not have a relationship with clinical healing and the amount of bone change over 12 months. Furthermore, the percent bone change significantly increased during the first three months.

**Conclusions:**

In conclusion, pre-operative factors, age and transplant site, influenced the healing rate of autogenous transplanted teeth. In contrast, the peri-operative factors were not related with the clinical and radiographic outcomes. Generating the least trauma to the periodontal ligament cells is the most important concern.

## Background

Autogenous tooth transplantation (ATT) provides various advantages over other treatment options. This treatment results in the esthetics of a natural tooth, is less expensive, and requires less treatment time. In young patients, ATT can be performed without interfering with jaw growth, unlike using a dental implant [1–5].

The recommended procedure [6, 7] is to extract a donor tooth and transplant it into a prepared recipient site in an atraumatic fashion. The amount of supporting bone can facilitate the healing and survival of the transplanted tooth [6]. However, delayed transplantation can result in alveolar bone resorption at the recipient site when the socket bone is extremely reduced to seat the donor tooth [8]. Although various clinical methods, including bone autograft and split osteotomy, have been suggested to promote bone regeneration in such cases [9, 10], these bone grafting techniques do not significantly accelerate or improve bone regeneration compared with alveolar bone preparation alone. Formation of the new alveolus depends on the vitality of the periodontal ligament (PDL) cells, which possess high potential for inducing bone tissue regeneration [9, 11].

There are many factors that affect bone healing after transplantation, such as the patient’s age, tooth condition, and operative approach [11–14]. Transplantation using a donor tooth that is larger than the edentulous space might jeopardize the PDL cells at the root surface. In contrast, teeth with an open root apex and less extra-alveolar time (EAT) are associated with a good ATT prognosis [14]. The techniques used during the operation, such as type of storage media and surgical trauma, clinically affect the preservation of the PDL cells on the root surface. Additionally, the amount of bone removed during preparation can lead to a prolonged healing process due to inflammation [2, 14].

Knowing what factors affect ATT success would be helpful for determining an appropriate treatment plan and determining the prognosis. Therefore, the aim of this study was to determine which factors were responsible for the ATT healing rate without the use of a bone graft at the recipient sites with insufficient bone support. The factors were classified as pre-operative and peri-operative and were analyzed in relation to the clinical and radiographic outcomes.

## Methods

### Patient selection

This retrospective study complied with the principles stated in the Declaration of Helsinki “Ethical Principles for Medical Research Involving Human Subjects”, adopted in 1964 and as amended in 2013, and was approved by the Ethics Committee of Mahidol University Institutional Review Board (COA.No.MU-DT/PY-IRB 2012/128.2607). We reviewed 151 ATT treatment records performed by the same surgeon at the Faculty of Dentistry, Mahidol University, between 1997–2007 with informed consent. Fifty records were included in this study. The exclusion criteria were failed cases (9 cases) due to infection related to poor oral care, lost to follow-up during the first year, and a lost radiograph (92 cases). The patients’ information comprised the operative record, radiographs, and status of the transplanted tooth. The follow-ups were performed at 1, 3, 6, and 12 months to observe wound healing, pulp, and periodontium regeneration.

### Treatment protocols

All cases were treated with the same surgeon using the same technique. The recipient site was prepared using stainless-steel round burs with a low-speed handpiece and saline irrigation under local anesthesia; 2% xylocaine with epinephrine 1:100,000 units. After recipient site preparation, atraumatic extraction of the donor tooth was performed using special care at the root surface. Adjusting the bone and trying-in the donor tooth into the prepared recipient area were done multiple times until the tooth fit. During the recipient site preparation, the donor tooth was kept in its socket or a mucoperiosteal pouch filled with blood to preserve the PDL cells. When the recipient site was adequately prepared, the donor tooth was placed into the prepared area below the adjacent occlusal plane to avoid occluding with the opposite tooth. The remaining buccal bone covering the root and the buccal gingiva covering the crown were measured. A cross-over suture was placed over the occlusal surface with a 4–0 Ethilon (Ethicon, UK) suture to stabilize the transplant, and this protocol was performed in every case. 1000 mg amoxicillin and 400 mg ibuprofen were given preoperatively, and, after the operation, 3 × 500 mg amoxicillin daily for a week were given together with 400 mg ibuprofen every 6 h prn. The patients were asked to avoid using the transplanted tooth and to eat a soft diet during the first month. The food consistency was increased if tolerable to the transplanted tooth.

### Data analysis

The information used in this study was divided into pre-, peri-, and post-operative data. The pre-operative factors were the age of the patients and the stages of root formation as described by Schwartz [15] (stage I: < 1/2 root length, stage II: 1/2–3/4 root length, stage III: > 3/4 root length with an open apex, and stage IV: 1/1 root length with a closed apex). In addition, the site of the donor tooth, the location of the recipient site (maxillary or mandibular), and the type of transplantation (intra-arch or inter arch) were analyzed. The peri-operative data comprised the EAT (min) during the surgery, the periodontium condition, the amount of bone removed (mm), and the residual bone and gingiva levels. In brief, the EAT was the time from donor tooth extraction until firmly seating it at the recipient site. The amount of bone removed was the difference between the pre- and post- surgical bone height from the CEJ of the adjacent tooth measured using a periodontal probe (Hu-Friedy, Chicago, USA). The remaining buccal bone was the post-surgical bone height, and the remaining buccal bone was categorized into three levels: no apical bone coverage, less than the apical 1/3, and at least at the apical 1/3. The gingival height covering the crown was grouped into three types: less than or equal to the cervical 1/3 of the crown, more than the cervical 1/3–2/3 of the crown, and more than 2/3 of the crown. Clinical and radiographic data were used to evaluate treatment outcome.

Gingival inflammation, tooth pain and mobility, and functional discomfort, were clinically observed and the duration that these signs and symptoms were present after surgery was recorded. The criteria were signs of redness and swollen gingiva for gingival inflammation, symptom of spontaneous pain and avoiding eating food at the transplantation site due to pain for tooth pain and functional discomfort respectively, and signs of tooth displacement in its socket using two handles of the instruments for investigating tooth mobility. Furthermore, the duration until the transplanted tooth responded to electrical pulp testing (EPT) was determined to evaluate dental pulp healing.

The radiographic outcomes were assessed using direct and indirect visualization of the periapical films taken using a standard procedure with the long-cone parallel technique. Direct visualization of the periapical films was performed by two examiners to detect bone healing, which was defined as complete trabeculation with a lamina dura [7] at each time interval, with Cohen’s kappa statistic as a measurement agreement between the two examiners (ĸ = 0.80; *P* < 0.01). Pulp obliteration was similarly examined to detect opacification of the pulp and blunting of the pulp horn [16, 17]. Indirect visualization was performed using digital subtraction to determine the alveolar bone change around the transplanted tooth. Image subtraction was done using ImageJ (NIH, USA) to measure the bone change in the region of interest (ROI) at the 3-, 6-, and 12-month follow-ups, as previously described [18–20]. Briefly, the images were realigned to a similar position using reference lines, which were the adjacent teeth’s CEJ in the images. The brightness and contrast were calibrated, and the threshold of the white-black pixel difference was set at the 256 greys level. The ROI surrounding the root of the transplanted tooth was created from the immediate post-operative radiograph at the area where bone was removed during recipient site preparation. The radiopacity was measured in the same ROI at each follow-up and represented as percentage per total area.

### Statistical analysis

The data were analyzed for a normal distribution and further statistical analysis using SPSS. The Chi-square test was used to identify correlations between the pre- and peri-operative factors and the clinical and radiographic outcomes. The T-test or one-way analysis of variance (ANOVA) was used to compare the percent bone change in each factor. A *P*-value < 0.05 was considered statistically significant.

## Results

Fifty successfully transplanted teeth were assessed in this study. The mean patient age was 19 ± 3.19 years old (13 males and 34 females). Most of the donor teeth were upper third molars (n = 32), while the remaining teeth comprised lower third molars (n = 16), an upper second molar (n = 1), and a lower second premolar (n = 1). The root formation of many of the donor teeth was in stage III (n = 33), and the others were in stage IV (n = 10), stage II (n = 6), and stage I (n = 1). Nine recipient sites were in the maxilla and 41 recipient sites were in the mandible, (24 intra-arch and 26 inter-arch transplantations). The mean EAT was 10.75 ± 10.26 min and the mean bone removal was 8.33 ± 3.39 mm.

**Fig. 1:**
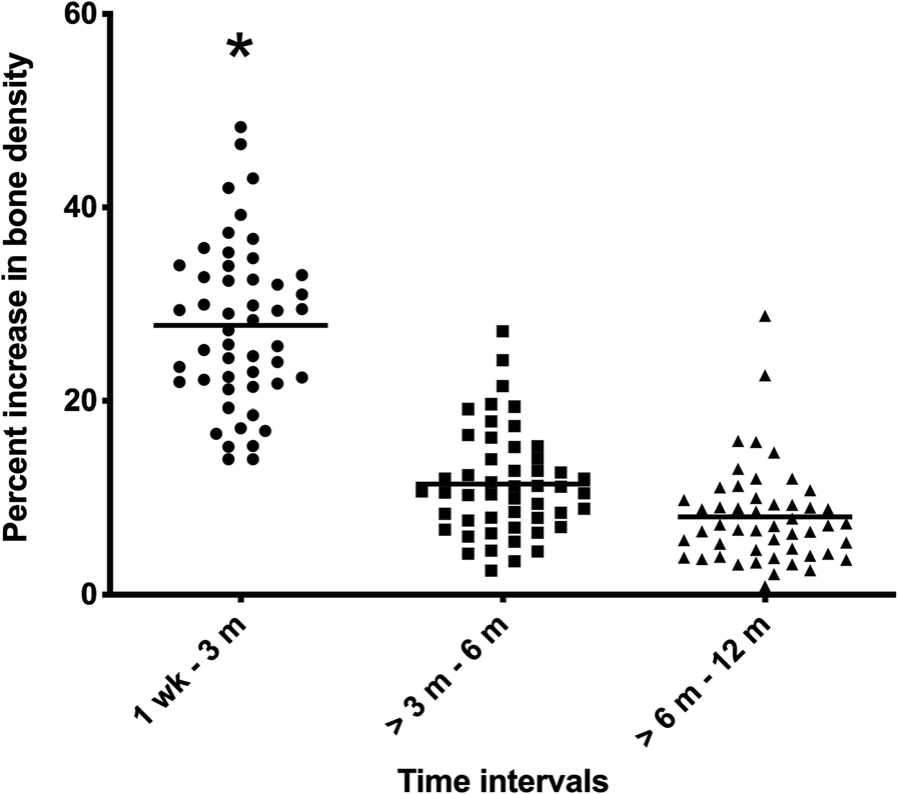
Percent increase in bone density. Percentage of bone change was evaluated at the 3-, 6-, and 12-month follow-ups. The change in radiopacity of the area surrounding the transplanted tooth was measured using subtraction radiography, *Significant at *p* < 0.05

The relationships between the pre- and peri-operative variables and the clinical and radiographic outcomes were analyzed. In general, the patients reported no spontaneous pain during the first week. Palpation and percussion responses were absent during the third month. During the first three months, a change in alveolar bone support was clinically detected in most teeth (> 60%) as showing a decline in tooth mobility from the 1st or 2nd degree after surgery to without signs of mobility or functional discomfort. The different ages, tooth parameters, and surgical approach did not demonstrate any relationship with the clinical signs and symptoms (Tables 1 and 2). However, the gingival healing at the intra-arch transplantation sites was delayed compared with the inter-arch transplantation sites (Chi-Square, df = 1; *P* < 0.05). The radiographic outcomes revealed that the surrounding bone in all treatments groups was completely regenerated, and the dental pulp was obliterated within 1 year. Further statistical analysis demonstrated that the recipient arch site was related to the duration for pulp obliteration to occur post-surgically (Chi-Square, df = 1; *P* < 0.05) (Table 3). Tremendous change in the radiopacity of the alveolar bone occurred during the first 3 months at 27 ± 8.37%, and then declined by 11.43 ± 5.41%, and 8.06 ± 5.11% after 6 and 12 months, respectively (Fig. 1). We found that the greatest amount of bone increase occurred in patients in the under 18-year-old group (ANOVA, F = 6.99; df = 1; *P* < 0.05) compared with the older aged group, while the peri-operative variables did not demonstrate a relationship with the amount of bone change over these periods (Table 4).

**Table 1:** Pre-operative and peri-operative factors related to the duration until no inflammation and the duration until no tooth mobility

		Time until no gingival inflammation: N (%)					Time until no tooth mobility: N (%)			
	Total (N)	1 wk–3 m	> 3–6 m	> 6–12 m	*p*-value		1 wk–3 m	> 3–6 m	> 6–12 m	*p*-value
*Age (years)*										
< 18	15	13 (86.7)	2 (13.3)	0	0.574		10 (66.7)	3 (20.0)	2 (13.3)	0.216
≥ 18	35	33 (94.3)	2 (5.7)	0			29 (82.9)	5 (14.3)	1 (2.9)	
*Donor tooth*										
Maxilla	33	32 (97.0)	1 (3.0)	0	0.108		26 (78.8)	4 (12.1)	3 (9.1)	0.295
Mandible	17	14 (82.4)	3 (17.6)	0			13 (76.5)	4 (23.5)	0	
*Recipient site*										
Maxilla	9	8 (88.9)	1 (11.1)	0	0.560		8 (88.9)	1 (11.1)	0	1
Mandible	41	38 (92.7)	3 (7.3)	0			31 (75.6)	7 (17.1)	3 (7.3)	
*Transplantation*										
Intra-arch	24	20 (83.3)	4 (16.7)	0	0.046*		19 (79.2)	5 (20.8)	0	0.260
Inter-arch	26	26 (100)	0	0			20 (76.9)	3 (11.5)	3 (11.5)	
*Root formation stage*										
Stage 1 + 2	7	7 (100)	0	0	1		7 (100)	0	0	0.194
Stage 3	33	30 (90.9)	3 (9.1)	0			22 (66.7)	8 (24.2)	3 (9.1)	
Stage 4	10	9 (90.0)	1 (10.0)	0			10 (100)	0	0	
*Extra-Alveolar time*										
< 15 min	27	24 (88.9)	3 (11.1)	0	1		21 (77.8)	5 (18.5)	1 (3.7)	1
≥ 15 min	15	14 (93.3)	1 (6.7)	0			11 (73.3)	3 (20.0)	1 (6.7)	
*Bone removal*										
< 8 mm	18	15 (83.3)	3 (16.7)	0	0.293		13 (72.2)	5 (27.8)	0	0.313
≥ 8 mm	25	24 (96.0)	1 (4.0)	0			20 (80.0)	3 (12.0)	2 (8.0)	
*Buccal plate covering the root*										
≥ 1/3	29	28 (96.6)	1 (3.4)	0	0.119		22 (75.9)	5 (17.2)	2 (6.9)	0.941
< 1/3	12	11 (91.7)	1 (8.3)	0			10 (83.3)	2 (16.7)	0	
No buccal plate	9	7 (77.8)	2 (22.2)	0			7 (77.8)	1 (11.1)	1 (11.1)	
*Buccal gingival covering the crown*										
> 2/3	17	16 (94.1)	1 (5.9)	0	0.431		12 (70.6)	4 (23.5)	1 (5.9)	0.911
(> 1/3–2/3)	20	19 (95.0)	1 (5.0)	0			16 (80.0)	3 (15.0)	1 (5.0)	
≤ 1/3	11	9 (81.8)	2 (18.2)	0			9 (81.8)	1 (9.1)	1 (9.1)	

^*^Statistical significance at *p* < 0.05

**Table 2:** Pre-operative and peri-operative factor related to the duration until normal function and the duration until a pulp response

		Time until normal function: N (%)				Time until pulp response: N (%)			
	Total (N)	1 wk–3 m	> 3-6 m	> 6–12 m	*p*-value	1 wk–3 m	> 3–6 m	> 6–12 m	*p*-value
*Age (years)*									
< 18	15	11 (73.3)	4 (26.7)	0	1	9 (60.0)	6 (40.0)	0	1
≥ 18	35	24 (68.6)	10 (28.6)	1 (2.9)		19 (54.3)	14 (40.0)	2 (5.7)	
*Donor tooth*									
Maxilla	33	24 (72.7)	9 (27.3)	0	0.467	19 (57.6)	12 (36.4)	2 (6.1)	0.685
Mandible	17	11 (64.7)	5 (29.4)	1 (5.9)		9 (52.9)	8 (47.1)	0	
*Recipient site*									
Maxilla	9	5 (55.6)	4 (44.4)	0	0.388	5 (55.6)	3 (33.3)	1 (11.1)	0.422
Mandible	41	30 (73.2)	10 (24.4)	1 (2.4)		23 (56.1)	17 (41.5)	1 (2.4)	
*Transplantation*									
Intra-arch	24	14 (58.3)	9 (37.5)	1 (4.2)	0.157	12 (50.0)	11 (45.8)	1 (4.2)	0.775
Inter-arch	26	21 (80.8)	5 (19.2)	0		16 (61.5)	9 (34.6)	1 (3.8)	
*Root formation stage*									
Stage 1 + 2	7	4 (57.1)	3 (42.9)	0	0.773	5 (71.4)	2 (28.6)	0	0.086
Stage 3	33	24 (72.7)	8 (24.2)	1 (3.0)		17 (51.5)	16 (48.5)	0	
Stage 4	10	7 (70.0)	3 (30.0)	0		6 (60.0)	2 (20.0)	2 (20.0)	
*Extra-Alveolar time*									
< 15 min	27	19 (70.4)	8 (29.6)	0	1	15 (55.6)	11 (40.7)	1 (3.7)	0.881
≥ 15 min	15	10 (66.7)	5 (33.3)	0		9 (60.0)	5 (33.3)	1 (6.7)	
*Bone removal*									
< 8 mm	18	14 (77.8)	4 (22.2)	0	0.713	13 (72.2)	4 (22.2)	1 (5.6)	0.330
≥ 8 mm	25	16 (64.0)	8 (32.0)	1 (4.0)		13 (52.0)	11 (44.0)	1 (4.0)	
*Buccal plate covering the root*									
≥ 1/3	29	21 (72.4)	7 (24.1)	1 (3.4)	0.856	18 (62.1)	10 (34.5)	1 (3.4)	0.645
< 1/3	12	8 (66.7)	4 (33.3)	0		6 (50.0)	5 (41.7)	1 (8.3)	
No buccal plate	9	6 (66.7)	3 (33.3)	0		4 (44.4)	5 (55.6)	0	
*Buccal gingival covering the crown*									
> 2/3	17	11 (64.7)	5 (29.4)	1 (5.9)	0.966	9 (52.9)	8 (47.1)	0	0.488
(> 1/3–2/3)	20	14 (70.0)	6 (30.0)	0		12 (60.0)	6 (30.0)	2 (10.0)	
≤ 1/3	11	8 (72.7)	3 (27.3)	0		5 (45.5)	6 (54.5)	0

**Table 3:** Pre-operative and peri-operative factors related to radiographic outcomes

		Time to complete trabeculation with lamina dura: N (%)				Time to pulp obliteration: N (%)			
	Total (N)	1 wk–3 m	> 3–6 m	> 6–12 m	*p*-value	1wk–3 m	> 3–6 m	> 6–12 m	*p*-value
*Age (years)*									
< 18	15	2 (13.3)	7 (46.7)	6 (40.0)	0.717	12 (80.0)	3 (20.0)	0	0.527
≥ 18	35	2 (5.7)	16 (45.7)	17 (48.6)		21 (60.0)	13 (37.1)	1 (2.9)	
*Donor tooth*									
Maxilla	33	2 (6.1)	14 (42.4)	17 (51.5)	0.505	23 (69.7)	9 (27.3)	1 (3.0)	0.685
Mandible	17	2 (11.8)	9 (52.9)	6 (35.3)		10 (58.8)	7 (41.2)	0	
*Recipient site*									
Maxilla	9	1 (11.1)	5 (55.6)	3 (33.3)	0.689	9 (100)	0	0	0.035*
Mandible	41	3 (7.3)	18 (43.9)	20 (48.8)		24 (58.5)	16 (39.0)	1 (2.4)	
*Transplantation*									
Intra-arch	24	3 (12.5)	12 (50.0)	9 (37.5)	0.349	17 (70.8)	7 (29.2)	0	0.876
Inter-arch	26	1 (3.8)	11 (42.3)	14 (53.8)		16 (61.5)	9 (34.6)	1 (3.8)	
*Root formation stage*									
Stage 1 + 2	7	0	3 (42.9)	4 (57.1)	1	4 (57.1)	3 (42.9)	0	0.88
Stage 3	33	3 (9.1)	15 (45.5)	15 (45.5)		22 (66.7)	10 (30.3)	1 (3.0)	
Stage 4	10	1 (10.0)	5 (50.0)	4 (40.0)		7 (70.0)	3 (30.0)	0	
*Extra-Alveolar time*									
< 15 min	27	1 (3.7)	11 (40.7)	15 (55.6)	0.203	18 (66.7)	8 (29.6)	1 (3.7)	1
≥ 15 min	15	0	10 (66.7)	5 (33.3)		10 (66.7)	5 (33.3)	0	
*Bone removal*									
< 8 mm	18	2 (11.1)	8 (44.4)	8 (44.4)	0.646	13 (72.2)	5 (27.8)	0	1
≥ 8 mm	25	1 (4.0)	13 (52.0)	11 (44.0)		17 (68.0)	7 (28.0)	1 (4.0)	
*Buccal plate covering the root*									
≥ 1/3	29	3 (10.3)	11 (37.9)	15 (51.7)	0.519	21 (72.4)	7 (24.1)	1 (3.4)	0.534
< 1/3	12	0	8 (66.7)	4 (33.3)		6 (50.0)	6 (50.0)	0	
No buccal plate	9	1 (11.1)	4 (44.4)	4 (44.4)		6 (66.7)	3 (33.3)	0	
*Buccal gingival covering the crown*									
> 2/3	17	3 (17.6)	5 (29.4)	9 (52.9)	0.386	11 (64.7)	5 (31.3)	1 (5.9)	0.91
(> 1/3–2/3)	20	1 (5.0)	11 (55.0)	8 (40.0)		13 (65.0)	7 (35.0)	0	
≤ 1/3	11	0	6 (54.5)	5 (45.5)		8 (72.7)	3 (27.3)	0	

^*^Statistical significance at *p* < 0.05

**Table 4:** Pre-operative and peri-operative factors related to bone change after transplantation

	Total (N)	1 wk–3 m		> 3–6 m		> 6–12 m			Total (N)	1wk–3 m	> 3–6 m	> 6–12 m
*Age (years)*								*EAT*				
< 18	15	32.1 ± 8.0		12.1 ± 6.5		8.73 ± 6.3		< 15 min	27	26.1 ± 8.5	11.6 ± 6.0	8.2 ± 5.4
≥ 18	35	25.8 ± 7.8		11.1 ± 4.9		7.7 ± 4.5		≥ 15 min	15	29.5 ± 7.1	10.5 ± 4.3	7.2 ± 3.8
*p*-value		0.011*		0.582		0.58		*p*-value		0.197	0.546	0.536
*Donor tooth*								*Bone removal*				
Maxilla	33	29.2 ± 8.2		11.5 ± 5.7		8.4 ± 5.7		< 8 mm	18	26.4 ± 8.6	10.5 ± 4.2	7.7 ± 5.2
Mandible	17	25.2 ± 8.3		11.2 ± 5.1		7.3 ± 3.6		≥ 8 mm	25	27.4 ± 7.9	10.9 ± 5.4	7.5 ± 3.5
*p*-value		0.112		0.851		0.484		*p*-value		0.697	0.807	0.863
*Recipient site*								*Buccal plate covering the root*				
Maxilla	9	29.1 ± 8.6		10.2 ± 3.0		8.3 ± 4.3		≥ 1/3 apical	29	28.0 ± 9.1	11.3 ± 4.5	7.4 ± 3.7
Mandible	41	27.5 ± 8.4		11.7 ± 5.8		8.0 ± 5.3		< 1/3 apical	12	26.2 ± 7.9	13.4 ± 7.6	10.0 ± 6.5
*p*-value		0.618		0.445		0.866		No buccal plate	9	29.4 ± 6.7	9.1 ± 3.9	7.6 ± 6.5
*Transplantation*								*p*-value		0.6777	0.191	0.425
Intra-arch	24	26.1 ± 8.6		10.9 ± 4.6		7.8 ± 3.9		*Buccal gingival covering the crown*				
Inter-arch	26	29.4 ± 8.0		11.9 ± 6.1		8.3 ± 6.1		> 2/3	17	26.6 ± 8.5	10.7 ± 5.0	7.4 ± 3.4
*p*-value		0.163		0.504		0.781		(> 1/3–2/3)	20	29.5 ± 9.8	11.5 ± 5.8	7.0 ± 4.2
*Stages of root formation*								≤ 1/3	11	26.3 ± 6.0	12.5 ± 6.2	10.8 ± 8.0
Stage 1 + 2	7	26.9 ± 7.8		12.0 ± 6.4		6.7 ± 1.1		*p*-value		0.476	0.709	0.092
Stage 3	33	28.4 ± 9.5		11.5 ± 5.8		8.6 ± 5.7						
Stage 4	10	26.6 ± 4.4		10.8 ± 3.2		7.0 ± 3.8						
*p*-value		0.686		0.838		0.415						

^*^Statistical significance at *p* < 0.05

## Discussion

The present study investigated the pre- and peri-operative variables that might affect the post-operative outcomes of ATT. We found that patient age and recipient arch type correlated with healing. In contrast, the peri-operative factors were not correlated with healing.

ATT with insufficient alveolar bone support might be possible without the use of a bone graft or other modalities. Our transplantations, despite the amount of bone removed during recipient site preparation and the remaining buccal plate covering the root, demonstrated similar clinical and radiographic outcomes. New bone formation was initially observed in our study within 3 months, which is earlier compared with Akiyama Y et al. [17]. Bauss O et al. [9] also demonstrated that transplanted teeth with or without using a bone graft exhibited similar success rates. Nine cases reported here lacked a buccal plate when transplanted, however, normal tooth function without signs of mobility was found in most teeth (6 teeth) at three months and all teeth within one year. Although the intraoral x-rays could not clearly display the buccal bone regeneration during the follow-ups, the findings in our previous case report [21] periapical films together with cone-beam computed tomography demonstrated the formation of a new buccal plate without using bone graft materials.

Typically, the amount of bone removed affects transplant stability, which could subsequently interfere with bone regeneration and pulp revascularization [22]. Although excessive buccal bone removal and little to no remaining bone support of the recipient site were noted, complete trabeculation with a lamina dura was seen in all cases after 12 months. The transplantations did not show signs of gingival inflammation or discomfort during function. Furthermore, our fixation step was carefully performed to obtain the optimal regenerative environment. The healing is better if ATT is fixed and secured from occlusal forces. We placed the transplanted tooth at least 1 mm below the occlusal plane during the healing phase based on Akiyama et al. [17]. Here, fixation was carefully performed to allow the ATT to heal normally during the early stage. Additional findings in this study demonstrated that the fixation allowed the surrounding soft tissue to be above the gingival margin to promote wound stability, because the level of the gingiva covering the crown after fixation did not influence inflammation.

The surrounding gingival healing in our cases was different between the inter-arch and intra-arch transplantations. The inter-arch transplantations healed more rapidly compared with the intra-arch, which is different from other studies [17, 21–23]. Previous reports found that in the intra-arch transplantations, the morphological fit between the donor tooth and recipient socket can lead to less trauma to the bone and soft tissue during socket preparation. However, our results might be due to the wound dimension. Most of the donor teeth and the recipient sites were close to each other in the intra-arch transplantations; thus, the soft tissue flap had to be wider and more aggressive compared with the inter-arch transplantations where a small soft tissue flap was made. Moreover, the size of the soft tissue flap may relate to the healing of the surrounding gingiva at the transplantation site if the oral hygiene care is inadequate.

Periodontal ligament cell vitality is a crucial aspect in the periodontal healing rate of the ATT, especially when there is inadequate alveolar support. Where the tooth is maintained during recipient site preparation is an important variable for the survival rate. Several studies [17, 24] have stored the donor tooth in a homeostasis balancing solution while the recipient site was prepared. However, there is no definitive conclusion of which chemical solution is the best choice. Alternatively, PDL cell preservation can be performed using the autogenous blood in the extracted donor tooth socket to preserve cell vitality [21] because plasma provides the appropriate pH and osmolarity for periodontal cells [23], and this agreed with our results that the duration that the extracted tooth was placed in the blood socket did not affect the pace of complete bone regeneration. Indeed, trauma to the tooth root during tooth placement is a major threat to PDL cell viability [23]. Thus, our findings suggest that extensive bone removal should be performed to reduce EAT and trauma to the transplanted tooth because the amount of remaining bone did not exhibit a relationship toward the rate of bone formation. Furthermore, the use of a computer-aided rapid prototype produced replica tooth might decrease the intra-operative time of recipient site preparation and the number of try-ins during donor tooth placement [25].

Bone change after transplantation was measured using a digital subtraction technique that quantitatively measured the opacification in the interested areas. The overall bone change in our study was the highest during the first 3 months, then slowly declined, similar to Waikakul et al. [7]. The operative variables did not affect the rate of new bone formation using this analysis method. Only the difference in age groups was related with the rate of bone regeneration during the first 3 months. The percent of bone increase in the patients under 18-years-old was faster compared with older patients, which agrees with previous studies [1, 24], where increased age negatively affected bone regeneration due to a reduced blood supply and re-mineralization rate. Another reason is the increase in periodontal diseases found in older patients [24].

Pulp canal obliteration is a sign of the pulp’s response to external stimuli and is a sign of pulp healing [26]. Likewise, electric pulp testing is a useful diagnostic tool, which extrapolates nerve innervation in the pulp. Both signs could be observed in our transplanted teeth during the follow-up within one year, suggesting that the surgical procedure results in and maintains pulp vitality. However, revascularization was not directly evaluated. A direct measurement of arterial blood flow can be performed using pulse oximetry, which many studies have used to evaluate pulp vitality [27]. In addition, the transplants in the maxillary recipient sites demonstrated significantly faster pulp obliteration compared with the mandible. A possible explanation is that the maxilla has an abundant blood supply, which is greater than in the mandible [28]. Our results also indicated that it was unnecessary in every ATT to perform endodontic treatment, which should be done when pulp necrosis or root resorption occurs as previously suggested [6]. Most of the teeth transplanted in the present study were immature permanent teeth, which are more prone to achieve revascularization. Only ten donor teeth presented with full root formation, which typically indicates a poor prognosis unless root canal treatment has been performed because high long-term survival (95%) and success (80%) rate were found when the transplanted mature tooth was endodontically treated [29]. A resected root tip can be an alternative option to enlarge the apical foramen for permitting revascularization of the mature teeth with complete root formation [30]. However, in our study we immediately transplanted the teeth without any additional procedure due to our transplants being young mature teeth with apical foramen that was open wide enough to allow for new vascular perfusion as previously reported [31–33]. Additionally, our previous case report demonstrated that the non-root canal treated tooth was vital at the 12-year follow-up [21]. However, in the present study, the patients were followed up for only 1 year. Further change and survival of the transplanted teeth might be different over a longer time. Additional studies with longer follow-up periods should be performed to evaluate the effect of these factors on ATT outcome.

## Conclusions

In conclusion, in recipient sites with insufficient bone support, all ATTs performed without a bone graft healed in terms of the periodontium, pulp, and function based on clinical and radiographic outcomes. ATT healing is not dependent on the amount of remaining buccal bone and gingiva. However, generating the least trauma to the periodontal ligament cells is the most important concern.

## Data Availability

The datasets used and analyzed during the current study are available from the corresponding author on reasonable request.

## Abbreviations

ATT: Autogenous tooth transplantation
PDL: Periodontal ligament
EAT: Extra-alveolar time
ANOVA: Analysis of variance
